# Cohort Differences in Well-Being Among Midlife and Older Women: Role of Self-Perceptions of Aging

**DOI:** 10.1093/geroni/igab046.214

**Published:** 2021-12-17

**Authors:** Lindsay Ryan

**Affiliations:** University of Michigan, Ann Arbor, Michigan, United States

## Abstract

The current study examines how cohort differences across two age-matched groups of midlife and older women from the Health and Retirement Study are associated with well-being and self-perceptions of aging (SPA). Women aged 51–60 (n=2318) and 61–70 (n=1650) were selected from the 2008 and 2018 waves. No significant cohort differences were identified for life satisfaction (Diener, Emmons, Larsen & Griffin, 1985) or positive SPA (Lawton, 1975; Liang & Bollen, 1983). The 2008 cohort of midlife women reported significantly higher negative SPA compared to 2018 (p<.05). Linear regression analyses find that cohort and SPA are significantly associated with life satisfaction in both age groups, and that the association of negative SPA differs by cohort for the midlife women (p<.01). Implications are discussed within the life course developmental framework.

